# Simple biochemical networks allow accurate sensing of multiple ligands with a single receptor

**DOI:** 10.1371/journal.pcbi.1005490

**Published:** 2017-04-14

**Authors:** Vijay Singh, Ilya Nemenman

**Affiliations:** 1 Department of Physics, Emory University, Atlanta, Georgia, United States of America; 2 Computational Neuroscience Initiative, University of Pennsylvania, Philadelphia, Pennsylvania, United States of America; 3 Department of Biology, Emory University, Atlanta, Georgia, United States of America; 4 Initiative in Theory and Modeling of Living Systems, Emory University, Atlanta, Georgia, United States of America; Rutgers University, UNITED STATES

## Abstract

Cells use surface receptors to estimate concentrations of external ligands. Limits on the accuracy of such estimations have been well studied for pairs of ligand and receptor species. However, the environment typically contains many ligands, which can bind to the same receptors with different affinities, resulting in cross-talk. In traditional rate models, such cross-talk prevents accurate inference of concentrations of individual ligands. In contrast, here we show that knowing the precise timing sequence of stochastic binding and unbinding events allows one receptor to provide information about multiple ligands simultaneously and with a high accuracy. We show that such high-accuracy estimation of multiple concentrations can be realized with simple structural modifications of the familiar kinetic proofreading biochemical network diagram. We give two specific examples of such modifications. We argue that structural and functional features of real cellular biochemical sensory networks in immune cells, such as feedforward and feedback loops or ligand antagonism, sometimes can be understood as solutions to the accurate multi-ligand estimation problem.

## Author summary

Cells live in chemically complex environments with many different chemical ligands around them. Can cells estimate concentrations of more ligands than they have receptor types? In this paper, we show that, surprisingly, the answer is “yes”, and the estimation can be implemented with simple biochemical components already present in many cells. Therefore, cells may “know” a lot more about their environment and thus may be able to implement more complex and accurate response strategies than was previously thought.

## Introduction

Cells obtain information about their environment by capturing ligand molecules with receptors on their surface and estimating the ligand concentration from the receptor activity. Limits on the accuracy of such estimation have been a subject of interest since the seminal work of Berg and Purcell [1], with several substantial extensions found recently [2–8]. Most of these assume one ligand species coupled to one receptor species, and the actual detection in most of these models is rather simple, involving counting the number or the duration of binding / unbinding events over a specific period of time.

However, cells carry many types of receptors and have many species of ligands around them. The same ligand can bind to many receptors, albeit with different affinities, and vice versa. This is commonly referred to as *cross-talk*. At the same time, real cellular sensory systems are incredibly complex, involving many dozens of identified biochemical species downstream of a typical receptor [9]. Functionally many of such signaling motifs are probably related to solving the cross-talk problem [10, 11], and are a topic of active research.

In traditional deterministic chemical kinetics, one cannot estimate concentrations of more ligands than there are receptor types. Further, even a weak cross-talk prevents determination of concentrations of individual chemical species since the activity of a receptor is a function of a weighted sum of concentrations of all ligands that can bind to it. In contrast, here we argue that, with cross-talk, concentration of more than one chemical species can be inferred from the activity of one receptor, provided that the stochastic temporal sequence of receptor binding and unbinding events is accessible instead of its mean occupancy. This is an important departure from the traditional view of cellular signaling that posits as many receptor types as there are ligand concentrations to be estimated. Indeed, previous works studying temporal sequences of receptor occupancy for ligand detection [11] and concentration estimation [5, 13, 12] have only considered the detection/estimation of a single ligand present in a mixture. We argue that the receptor occupancy sequence contains much more information about the mixture. In fact, based on the maximum likelihood techniques, which have been used previously to study receptor occupancy, we show that *all* components of the ligand mixture can be estimated by just one receptor, at least in principle. This surprising result can be understood by noting that a typical duration of time that a ligand remains bound to the receptors depends on its unbinding rate. Thus observing the statistics of the receptor’s *unbound* time durations allows estimation of a weighted average of all chemical species that interact with it [5]. Then the statistics of the *bound* time durations tells how common each ligand is.

The result is very general and independent on the choice of a downstream biochemical kinetics scheme that actually performs the estimation. In this article, we derive the result for the simplest problem of this class, namely one receptor interacting with two ligand species. While the exact solution of the inference problem for finding both ligand concentrations is hard to implement using common biochemical machinery, we show that an accurate approximation is possible using simple extensions of the familiar kinetic proofreading mechanism [14, 15]. We identify examples of such motifs implementing such estimation of multiple concentrations in signaling networks found downstream of many immune receptors [9], arguing that real biological systems may be implementing such multivariate concentration sensing. The kinetic schemes that we analyze detect rare ligands more accurately than a simple kinetic proofreading does, and we argue that the involved biochemical computation can explain properties like ligand antagonism, commonly observed in receptor signaling.

Overall, these different arguments support our main idea, that *the temporal sequence of binding and unbinding on a single receptor can provide an accurate estimate of the concentration of multiple ligands that bind to the receptor, and that the involved calculations can be performed reliably by known biochemical networks.*

## Results

### The model

**Fig 1:**
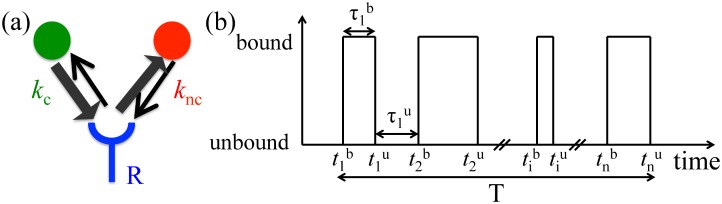
The model. (a). Two ligands, cognate and non-cognate having concentrations *c*_c_ and *c*_nc_, bind to a receptor R with binding rates *k*_c_ and *k*_nc_, respectively. The cognate unbinding rate is defined as lower than the non-cognate one (*r*_c_ < *r*_nc_). (b) Time series of receptor occupancy is used to determine both on-rates.

Consider a single receptor interacting with a cognate and a non-cognate ligand (Fig 1) that have the concentrations *c*_c_ and *c*_nc_, respectively. The binding rate of the ligands to the receptor are *k*_c_ and *k*_nc_. The binding rates are diffusion limited and hence *k*_c_∼*k*_nc_. It is the unbinding or off-rates, *r*_c_ and *r*_nc_, that distinguish the two ligands: *r*_nc_ > *r*_c_, and a cognate molecule typically stays bound for longer. The binding and unbinding rates (*k*_*α*_’s and *r*_*α*_’s) are fixed and can be assumed known for each receptor-ligand pair. Thus we are interested in the estimation of the ligand concentrations only, *c*_c_ and *c*_nc_. Following Ref. [5], we estimate *c*_c_ and *c*_nc_ from the time-series of binding, $\left\{t_{i}^{b}\right\}$, and unbinding, $\left\{t_{i}^{u}\right\}$, events of a total duration *T* using Maximum Likelihood techniques, paralleling a recent similar independent discussion, which focused on detection of a single ligand concentration [12]. The numbers of binding and unbinding events are different by, at most, one, which is insignificant since we consider *T* → ∞. Thus without loss of generality, we assume that the first event was a binding event at $t_{1}^{b}$, and the last one was the unbinding at $t_{n}^{u}$. We write the probability distribution of observing the sequence $\left\{t_{1}^{b},t_{1}^{u},\ldots ,t_{n}^{b},t_{n}^{u}\right\}$, or alternatively the sequence of binding and unbinding intervals $\tau_{i}^{b}=t_{i}^{u}-t_{i}^{b}$, and $\tau_{i}^{u}=t_{i+1}^{b}-t_{i}^{u}$:

$$P\equiv P\left(\left\{\tau_{i}^{b},\;\tau_{i}^{u}\right\}|c_{c},c_{\text{nc}}\right)=\frac{1}{Z}\prod_{i=1}^{n}\left[e^{-\tau_{i}^{u}\left(k_{c}c_{c}+k_{\text{nc}}c_{\text{nc}}\right)}\left(k_{c}c_{c}\;r_{c}\;e^{-\tau_{i}^{b}r_{c}}+k_{\text{nc}}c_{\text{nc}}\;r_{\text{nc}}\;e^{-\tau_{i}^{b}r_{\text{nc}}}\right)\right].$$ (1)

Here the first term under the product sign is the probability of the receptor staying unbound for $\tau_{i}^{u}$. The second term, which from now on we denote by $D(c_{c},c_{\text{nc}},\tau_{i}^{b})$, is proportional to the probability of staying bound for $\tau_{i}^{b}$. $D(c_{c},c_{\text{nc}},\tau_{i}^{b})$ has contributions from binding events from both the cognate and the noncognate ligands, with odds of *c*_c_ and *c*_nc_, respectively. Finally, *Z* is the normalization,

$$Z=\sum P(\left\{\tau_{i}^{b},\;\tau_{i}^{u}\right\}|c_{c},c_{\text{nc}}),$$ (2)

where the sum is over all sequences of duration *T* and *n* binding-unbinding events. Note that here we define $\tau_{n}^{u}=t_{1}^{b}+\left(T-t_{n}^{u}\right)$, so that the *n*’th unbound interval includes the “incomplete” unbound intervals before the first binding and after the last unbinding.

The log-likelihood of *c*_c_ and *c*_nc_ is the logarithm of *P*, Eq (1). Taking the derivatives of the log-likelihood w. r. t. *c*_c_ and *c*_nc_ and setting them to zero gives the Maximum Likelihood (ML) equations for the concentrations. Denoting by $T^{u}=\sum_{i=1}^{n}\tau_{i}^{u}$, the total time the receptor is unbound, these ML equations are (see Methods for the derivation):

$$-k_{c}T^{u}+\sum_{i=1}^{n}\frac{k_{c}r_{c}e^{-\tau_{i}^{b}r_{c}}}{D(c_{c}^{*},c_{\text{nc}}^{*},\tau_{i}^{b})}=0,$$ (3)

$$-k_{\text{nc}}T^{u}+\sum_{i=1}^{n}\frac{k_{\text{nc}}r_{\text{nc}}e^{-\tau_{i}^{b}r_{\text{nc}}}}{D(c_{c}^{*},c_{\text{nc}}^{*},\tau_{i}^{b})}=0,$$ (4)

where $c_{c}^{*}$ and $c_{\text{nc}}^{*}$ denotes the ML solution. Multiplying Eqs (3) and (4) by $c_{c}^{*}$ and $c_{\text{nc}}^{*}$, respectively, and adding them gives

$$k_{c}c_{c}^{*}+k_{\text{nc}}c_{\text{nc}}^{*}=\frac{n}{T^{u}}.$$ (5)

As in Ref. [5], the total on-rate (the weighted average of the external concentrations) is determined only by the average duration of the unbound interval, (*n*/*T*^u^)^−1^, because no binding is possible when the receptor is already bound. For the special case of *k*_c_ ≈ *k*_nc_ ≈ *k* (for ligands with binding rate determined by diffusion), Eq (5) determines the maximum likelihood estimate of the sum of the two concentrations, similar to the result in Ref. [5, 12]:

$$c_{\text{tot}}^{*}=\left(c_{c}^{*}+c_{\text{nc}}^{*}\right)=\frac{n}{T^{u}k}.$$ (6)

This shows that the estimates are negatively correlated. For general *k*_*i*_’s, a weighted sum of the concentrations is determined, but the negative correlation persists.

To get the individual concentrations, we need to solve the ML equations Eqs (3) and (4). In general, they can only be solved numerically. However, as all ML estimators, they are unbiased to the leading order in *n* (we verified this numerically). The standard errors of the ML estimates can be obtained by inverting the Hessian matrix,

$${\left.\frac{\partial^{2}\log P}{\partial c_{\alpha }\partial c_{\beta }}\right|}_{c_{c}^{*},c_{\text{nc}}^{*}}=\sum_{i=1}^{n}\left[\frac{-1}{D{\left(c_{c},c_{\text{nc}},\tau_{i}^{b}\right)}^{2}}\times \left(\begin{array}{cc} k_{c}^{2}r_{c}^{2}e^{-2\tau_{i}^{b}r_{c}} & k_{c}k_{\text{nc}}r_{c}r_{\text{nc}}e^{-\tau_{i}^{b}\left(r_{c}+r_{\text{nc}}\right)}\\ k_{c}k_{\text{nc}}r_{c}r_{\text{nc}}e^{-\tau_{i}^{b}\left(r_{c}+r_{\text{nc}}\right)} & k_{\text{nc}}^{2}r_{\text{nc}}^{2}e^{-2\tau_{i}^{b}r_{\text{nc}}} \end{array}\right)\right],$$ (7)

where greek indices stand for {c, nc}. Each term in the Hessian matrix is a sum of *n* numbers, each smaller than zero. The inverse of $\frac{\partial^{2}\log P}{\partial c_{\alpha }\partial c_{\beta }}$, which scales as ∝ 1/*n*, sets the minimum variance of any unbiased estimator according to the Cramer-Rao bound. It has straightforward analytical approximations in various regimes. For example, when the noncognate ligand is almost absent (*c*_c_/*c*_nc_ ≫ 1), and its few molecules do not bind for long (*r*_c_/*r*_nc_ ≪ 1), one gets $\sigma^{2}\left(c_{c}^{*}\right)\approx {\left(\partial^{2}\log P/\partial c_{c}^{2}\right)}_{c_{c}=c_{c}^{*}}^{-1}\approx 1/n$, matching the accuracy of sensing one ligand with one receptor [5]. A regime relevant for detection of a rare, but highly specific ligand [11, 12, 16] can be investigated as well. For now, we focus on how the receptor estimates (rather than detects) concentrations of *both* ligands simultaneously, which requires us to explore the full range of on- and off-rates.

**Fig 2:**
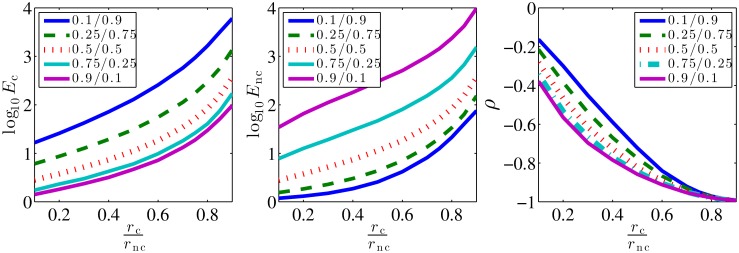
Variability of the ML estimators, represented by log_10_ *E*_c_ (left), log_10_ *E*_nc_ (center), and the correlation coefficient *ρ* between $c_{\text{c}}^{*}$ and $c_{\text{nc}}^{*}$ (right) as functions of the ratio of unbinding rates. Here we use *r*_nc_ = 1, *k*_c_ = *k*_nc_ = 1, *c*_c_ + *c*_nc_ = 1. The legend and the colors represent different ratios of concentrations of the cognate and the non-cognate ligands $\left(\frac{c_{c}}{c_{\text{nc}}}\right)$. We plot averages over 30,000 randomly generated binding/unbinding sequences for each combination of the rates. Each sequence itself consists of *n* = 30,000 binding events, simulated using the Gillespie algorithm. Standard errors are too small to show.

The estimates of the concentration *c*_c_ and *c*_nc_ are obtained by numerically solving ML equations, Eqs (3) and (4). We study the variability of these ML estimators in terms of their posterior variances. Notice that these posterior variances scale as 1/*n*, so we define the error of the ML estimators, *E*, as the squared coefficient of variation times the number of binding-unbinding events, *n*. Hence, we have, $E_{c}=n\sigma^{2}\left(c_{c}^{*}\right)/c_{c}^{2}$ and $E_{\text{nc}}=n\sigma^{2}\left(c_{\text{nc}}^{*}\right)/c_{\text{nc}}^{2}$ for cognate and non-cognate ligands, respectively. These quantities have a finite limit at *n* → ∞. Specifically, *E* = 1 is the accuracy that a receptor that binds only a single ligand can obtain [5]. Thus *E*_c_ and *E*_nc_ compare the performance of our multi-ligand ML estimator to the limit achievable by a single ligand ML estimator. We show log_10_ *E*_c_ and log_10_ *E*_nc_ for different concentrations and off-rates in Fig 2. If the two ligands are readily distinguishable, *r*_c_ ≪ *r*_nc_, then the ligand with the larger concentration has *E* ∼ 1. When *c*_c_ ∼ *c*_nc_, *E*_i_ ∼ 4…5, and it grows to 10…30 for a ligand with a very small relative concentration. Emphasizing the importance of the time scale separation, *E* > 100 if the ligands are hard to distinguish, *r*_c_ ∼ *r*_nc_. Here the correlation coefficient *ρ* of the two estimates reaches −1 because the same binding event can be attributed to either ligand. Finally, the asymmetry of the plots w. r. t. the exchange of *c*_c_ and *c*_nc_ is because the cognate ligand can generate short binding events, while long events from the noncognate ligand are exponentially unlikely. In summary, it is possible to infer two ligand concentrations from one receptor, with the error of only 1…10 times larger than for ligand-receptor pairs with no cross talk, as long as the two off-rates are substantially different. This complements the findings of [12] that a single concentration can be inferred from a time series of “on” and “off” events in a background of noncognate bindings using Maximum Likelihood estimation. We have verified that the analytical expression for the estimation error derived in Ref. [12] for a single cognate ligand matches our numerical results (see Methods).

### Approximate solution

It is not clear if there exist biochemical networks that can solve the ML equations, Eqs (3) and (4), exactly. Luckily, an approximate solution exists. Note that most of the long binding events come from the cognate ligand since the noncognate one dissociates faster. Defining long events as $\tau_{i}^{b}\geq T^{c}$ and using Eq (5), we rewrite Eq (3) as

$$\frac{k_{c}n}{k_{c}c_{c}^{*}+k_{\text{nc}}c_{\text{nc}}^{*}}=\left(\sum_{\tau_{i}^{b}\geq T^{c}}+\sum_{\tau_{i}^{b}<T^{c}}\right)\frac{k_{c}r_{c}e^{-\tau_{i}^{b}r_{c}}}{D(c_{c}^{*},c_{\text{nc}}^{*},\tau_{i}^{b})}$$ (8)

Assuming that all long events are cognate, *T*^c^ ≫ 1/*r*_nc_, gives

$$\frac{k_{c}n}{\left(k_{c}c_{c}^{a}+k_{\text{nc}}c_{\text{nc}}^{a}\right)}=\frac{n_{l}}{c_{c}^{a}}+\sum_{\tau_{i}^{b}<T^{c}}\frac{k_{c}r_{c}e^{-\tau_{i}^{b}r_{c}}}{D(c_{c}^{a},c_{\text{nc}}^{a},\tau_{i}^{b})},$$ (9)

where *n*_l_ is the number of long events, and the superscript “a” stands for the *a*pproximate solution. If further *T* is long enough so that there are many short events, and a single binding duration hardly affects $k_{c}^{*}$, then the sum in Eq (9) can be approximated by the expectation value:

$$\frac{n}{\left(k_{c}c_{c}^{a}+k_{\text{nc}}c_{\text{nc}}^{a}\right)}=\frac{n_{l}}{k_{c}c_{c}^{a}}+\left(n-n_{l}\right)\int_{0}^{T_{c}}\frac{r_{c}e^{-\tau^{b}r_{c}}P\left(\tau^{b}|c_{c}^{a},c_{\text{nc}}^{a}\right)d\tau^{b}}{D(c_{c}^{a},c_{\text{nc}}^{a},\tau^{b})},$$ (10)

where $P(\tau^{b}|c_{c}^{a},c_{\text{nc}}^{a})$ is the probability of observing a binding event of duration *τ*^b^ for the given binding rates,

$$P\left(\tau^{b}|c_{c}^{a},c_{\text{nc}}^{a}\right)=\frac{D(c_{c}^{a},c_{\text{nc}}^{a},\tau^{b})}{\left(k_{c}c_{c}^{a}+k_{\text{nc}}c_{\text{nc}}^{a}\right)}.$$ (11)

Plugging Eq (11) into Eq (10), we obtain

$$\frac{1}{\left(k_{c}c_{c}^{a}+k_{\text{nc}}c_{\text{nc}}^{a}\right)}=\frac{n_{l}}{nk_{c}c_{c}^{a}}+\left(1-\frac{n_{l}}{n}\right)\frac{1-e^{-r_{c}T^{c}}}{\left(k_{c}c_{c}^{a}+k_{\text{nc}}c_{\text{nc}}^{a}\right)}.$$ (12)

Finally, since *n*_l_ ≪ *n*, using Eq (5), we get (see Methods for a detailed derivation):

$$c_{c}^{a}=\frac{1}{k_{c}}\frac{n_{l}}{T^{u}}e^{r_{c}\;T^{c}},$$ (13)

$$c_{\text{nc}}^{a}=\frac{1}{k_{\text{nc}}}\left(\frac{n}{T^{u}}-\frac{n_{l}}{T^{u}}e^{r_{c}\;T^{c}}\right).$$ (14)

In other words, the approximate cognate ligand concentration is proportional to the number of long events.

We can estimate the bias and the variance of $c_{c}^{a}$ and $c_{\text{nc}}^{a}$ in a limiting case. If *r*_c_ and *r*_nc_ are not very different from each other, then one needs to focus on extremely long events in order to identify cognate bindings. This is only possible if *T*^c^ is much larger than the inverse of both of the unbinding rates, $T^{c}\gg \left\{r_{\text{nc}}^{-1},r_{c}^{-1}\right\}$. Large *T*^c^ ensures that the long binding events get no or minimal contribution from non-cognate ligands. However, since the time for which the receptor stays bound is exponentially distributed, under this condition, the number of “long” events (such that *τ_b_* > *T*^c^) would be very small, *n*_l_ ≪ *n*. Thus most of the variance of $c_{c}^{a}$ and $c_{\text{nc}}^{a}$ in Eqs (13) and (14) comes from the variability of *n*_l_, but not *T*^u^ (since *T*^u^ ∝ *n*). Thus we write $\langle c_{c}^{a}\rangle \approx \frac{\langle n_{l}\rangle }{\langle T^{u}\rangle }\frac{e^{r_{c}T^{c}}}{k_{c}}$. Further, the individual unbound periods are independent, so that 〈*T*^u^〉 = *n*〈*τ*^u^〉 = *n*/(*k*_c_*c*_c_ + *k*_nc_*c*_nc_) (notice the use of *c* rather than *c*^a^ here). Further, $\langle n_{l}\rangle =n\;P\left(\tau^{b}>T^{c}\right)=\frac{n}{\left(k_{c}c_{c}+k_{\text{nc}}c_{\text{nc}}\right)}(k_{c}c_{c}e^{-r_{c}T^{c}}+k_{\text{nc}}c_{\text{nc}}e^{-r_{\text{nc}}T^{c}})$. Combining these expressions, we get

$$\langle c_{c}^{a}\rangle \approx c_{c}+\frac{k_{\text{nc}}c_{\text{nc}}}{k_{c}}e^{-\left(r_{\text{nc}}-r_{c}\right)T^{c}}.$$ (15)

Thus for large *T*^c^, the bias of the approximate estimator, $\frac{k_{\text{nc}}c_{\text{nc}}}{k_{c}}e^{-\left(r_{\text{nc}}-r_{c}\right)T^{c}}$, grows with the relative number of noncognate long bindings events. In turn, the latter is proportional to *c*_nc_, but decreases exponentially with *T*^c^.

Within the same approximation, the variance of the estimator is given by $\sigma^{2}\left(c_{c}^{a}\right)\approx \frac{\sigma^{2}\left(n_{l}\right)}{{\langle T^{u}\rangle }^{2}}\frac{e^{2r_{c}T^{c}}}{k_{c}^{2}}$. However, long binding events are rare, independent of each other, and hence obey the Poisson statistics. Thus *σ*^2^(*n*_l_) = 〈*n*_l_〉, so that

$$\sigma^{2}\left(c_{c}^{a}\right)\approx \langle c_{c}^{a}\rangle \;\frac{\left(c_{c}+k_{\text{nc}}c_{\text{nc}}/k_{c}\right)}{n}e^{r_{c}T^{c}}.$$ (16)

The variance obviously grows with *T*^c^.

Knowing that the bias and the variance of the approximation change in opposite directions with *T*^c^, we can find the optimal cutoff ($T_{*}^{c}$) by minimizing the overall error. We define such error *L* as the sum of the variance and the squared bias of the estimator, i. e.,

$$L_{c}=\left[\sigma^{2}\left(c_{c}^{a}\right)+{\left(c_{c}-\langle c_{c}^{a}\rangle \right)}^{2}\right],$$ (17)

$$L_{\text{nc}}=\left[\sigma^{2}\left(c_{\text{nc}}^{a}\right)+{\left(c_{\text{nc}}-\langle c_{\text{nc}}^{a}\rangle \right)}^{2}\right].$$ (18)

The optimal cutoff is obtained by minimizing *L*_c_ or, in other words, solving the bias-variance tradeoff:

$$T_{*}^{c}=\arg\min_{T^{c}}L_{c}.$$ (19)

Near the optimal cutoff, the bias is small, and we use *c*_c_ instead of $c_{c}^{a}$ for the variance of the estimator, Eq (16). Then solving Eq (19) gives:

$$T_{*}^{c}=\frac{1}{\left(2r_{\text{nc}}-r_{c}\right)}\log\left[2T^{u}\left(\frac{r_{\text{nc}}}{r_{c}}-1\right)\frac{k_{\text{nc}}^{2}c_{\text{nc}}^{2}}{k_{c}c_{c}}\right].$$ (20)

Plugging this into Eqs (15) and (16), we get the minimal error of the estimator, which we omit here for brevity.

**Fig 3:**
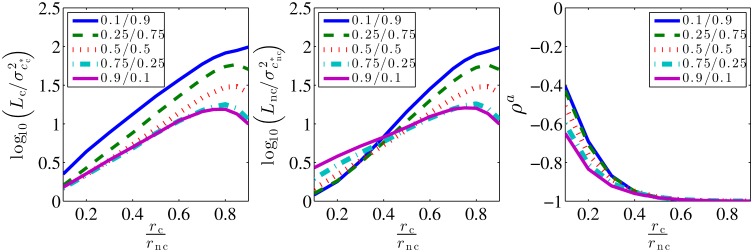
Comparison of errors of the approximate and the ML solutions. We plot $\log_{10}\left(L_{c}\left(T_{0}\right)/\sigma_{c_{c}^{*}}^{2}\right)$ (left), $\log_{10}\left(L_{\text{nc}}\left(T_{0}\right)/\sigma_{c_{\text{nc}}^{*}}^{2}\right)$ (center) and the covariance of the approximate estimates (right) as functions of on- and off-rates. Simulations are performed in the same way as in Fig 2. Legends and color scheme are the same as in Fig 2.

The optimal cutoff is proportional to 1/*r*_nc_ if *r*_nc_ ≫ *r*_c_, and it grows with *r*_c_, allowing for better disambiguation of cognate and noncognate events. Crucially, the off-rates are dictated by the ligand identities. In contrast, the concentrations, *c*_c_ and *c*_nc_, are what the receptors measures. Therefore, it is encouraging that $T_{*}^{c}$ depends only logarithmically on the concentrations (and also on the duration of the measurement, *T*^u^). Thus even if *T*^c^ is fixed as $T_{*}^{c}$ at some fixed values of *c*_c_, *c*_nc_, it remains near-optimal for a broad range of external concentrations. To illustrate this, we use $T^{c}=T_{*}^{c}\left(k_{c}c_{c}=k_{\text{nc}}c_{\text{nc}}=1/2\right)\equiv T_{0}$ and analyze the quality of the approximation in Fig 3, where we plot the ratio $L_{c}\left(T_{0}\right)/\sigma_{c_{c}}^{2}$ and $L_{\text{nc}}\left(T_{0}\right)/\sigma_{c_{\text{nc}}}^{2}$. Notice that $\sigma_{c_{c}}^{2}$ and $\sigma_{c_{\text{nc}}}^{2}$, the variances of the exact ML estimators, are proportional to *E*_c_ and *E*_nc_, respectively. Since ML estimators are unbiased, the ratios $L_{c}\left(T_{0}\right)/\sigma_{c_{c}}^{2}$ and $L_{\text{nc}}\left(T_{0}\right)/\sigma_{c_{\text{nc}}}^{2}$ compare the errors of the approximate solution to the errors *E*_c_ and *E*_nc_. Since these ratios approach 1 when *r*_c_/*r*_nc_ → 0 (specifically, for *r*_c_/*r*_nc_ = 0.1, $L_{c}\left(T_{0}\right)/\sigma_{k_{c}}^{2}\approx 1.47$, and $L_{\text{nc}}\left(T_{0}\right)/\sigma_{k_{\text{nc}}}^{2}\approx 1.21$), we conclude that the approximation is accurate even at fixed *T*^c^ = *T*_0_ when its assumptions are satisfied. This happens even though $T_{*}^{c}$ depends on *c*_c_ and *c*_nc_, but apparently the approximate estimates are as good as the ML estimates even at fixed *T*^c^ = *T*_0_ and work well for a large range of concentration ratios. This is important, as the molecular mechanisms that sets the delays in the cell does not need to be modified for different ligand concentrations.

In contrast, when the ligands are nearly indistinguishable (*r*_c_/*r*_nc_ ∼ 1), both $L_{c}\left(T_{0}\right)/\sigma_{k_{c}}^{2}∼100$ and $L_{\text{nc}}\left(T_{0}\right)/\sigma_{k_{\text{nc}}}^{2}∼100$, but here one would not use one receptor to estimate two concentrations since even the ML solution is bad (cf. Fig 2). Note also that both *L*_c_ and *L*_nc_ are smaller for *r*_c_ ∼ *r*_nc_ if *c*_c_ ≫ *c*_nc_. This is because our main assumption (that almost all long events are cognate) holds better when cognate ligands dominate. Finally, the correlation coefficient between the approximate estimates, *ρ*^a^ (right panel) reaches -1 earlier than in Fig 2. This is a direct consequence of Eqs (13) and (14).

### Kinetic proofreading for approximate estimation

The approximate solution can be computed by cells using the well-known kinetic proofreading (KPR) mechanism [14, 15, 17, 18]. In the simplest model of KPR [19], intermediate states between an inactive and an active state of a receptor delay the activation. Thus bound ligands can dissociate before the receptor activates, at which point it quickly reverts to the inactive state. Since *r*_c_ < *r*_nc_, cognate ligands dominate among bindings that persist to activation. The resulting increase in specificity in various KPR schemes has led to their exploration in the context of *detection* of rare ligands [11, 12, 16, 18]. Instead, here we analyze their ability to *measure* concentrations of both ligands simultaneously. We first consider the case where both the cognate and the non-cognate ligand concentration are comparable, *c*_c_ ∼ *c*_nc_ and the dissociation rates are distinct, *r*_c_ ≪ *r*_nc_. In the following sections, we explore another case, *c*_c_ ≪ *c*_nc_ and *r*_c_ ≲ *r*_nc_, a situation common in immunology.

**Fig 4:**
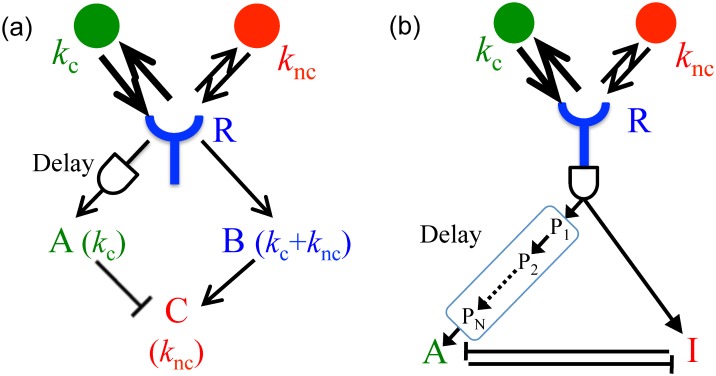
Kinetic proofreading for estimating multiple concentrations. Two different kinetic schemes that allow using kinetic proofreading for estimation of chemical concentrations. (A) Molecules A and B are produced when the receptor R is bound, but A is produced only for long bindings, implemented through the KPR delay. Another chemical species C subtracts A from B, so that A approximates *c*_c_ and C approximates *c*_nc_. This kinetic scheme would simultaneously estimate relatively similar concentrations of two ligands with substantially distinct off-rates *r*_c,nc_. (B) Molecules A and I (inhibitor of A) are produced when the receptor is bound (possibly after a KPR delay). Production of A is delayed even further, as it results at the end of a sequence of intermediate products P_i_. A and I bind to each other nearly irreversibly, sequestering and deactivating each other. The first delay at the receptor filters very short, nonspecific bindings. Because of the additional KPR delay on the A branch, A and I branches are then different linear combinations of strongly (cognate) and medium (non-cognate) binding ligands. If various kinetic parameters are tuned (see text), then even for very similar *r*_c_ and *r*_nc_, and even for a rare cognate ligand, the sequestration can remove the non-cognate contribution from the A branch.

Consider a biochemical network in Fig 4(a): the receptor, R, activates two messenger molecules, A and B. The former is activated with the rate *k*_A_ only if the receptor stays bound for longer than a certain *T*^c^ (with the delay achieved using the KPR intermediate states). The latter is activated with the rate *k*_B_ whenever the receptor is bound. The molecules deactivate with the rates *r*_A_ and *r*_B_, respectively, and all activations/deactivations are first-order reactions. Then the mean concentrations of the messenger molecules are (see Methods):

$$\bar{A}=\frac{k_{c}c_{c}/r_{c}e^{-r_{c}T^{c}}+k_{\text{nc}}c_{\text{nc}}/r_{\text{nc}}e^{-r_{\text{nc}}T^{c}}}{1+k_{c}c_{c}/r_{c}+k_{\text{nc}}c_{\text{nc}}/r_{\text{nc}}}\frac{k_{A}}{r_{A}},$$ (21)

$$\bar{B}=\frac{k_{c}c_{c}/r_{c}+k_{\text{nc}}c_{\text{nc}}/r_{\text{nc}}}{1+k_{c}c_{c}/r_{c}+k_{\text{nc}}c_{\text{nc}}/r_{\text{nc}}}\frac{k_{B}}{r_{B}}.$$ (22)

Assuming again that most bindings longer than *T*^c^ are cognate (*T*^c^ ≫ 1/*r*_nc_), Eq (21), can be written as:

$$\bar{A}=\frac{k_{c}c_{c}/r_{c}e^{-r_{c}T^{c}}}{1+k_{c}c_{c}/r_{c}+k_{\text{nc}}c_{\text{nc}}/r_{\text{nc}}}\frac{k_{A}}{r_{A}}.$$ (23)

Further, it is easy to see that Eq (22) can be rewritten as:

$$\frac{k_{c}c_{c}}{r_{c}}+\frac{k_{\text{nc}}c_{\text{nc}}}{r_{\text{nc}}}=\frac{\bar{B}}{k_{B}/r_{B}-\bar{B}}.$$ (24)

Now solving Eqs (23) and (24) for the on-rates, we get

$$c_{c}=\frac{\bar{A}e^{r_{c}T^{c}}r_{c}r_{A}}{k_{c}k_{A}}\left(1+\frac{\bar{B}}{k_{B}/r_{B}-\bar{B}}\right),$$ (25)

$$c_{\text{nc}}=\frac{r_{\text{nc}}}{k_{\text{nc}}}\left[\frac{\bar{B}}{k_{B}/r_{B}-\bar{B}}-\frac{\bar{A}e^{r_{c}T^{c}}r_{A}}{k_{A}}\left(1+\frac{\bar{B}}{k_{B}/r_{B}-\bar{B}}\right)\right].$$ (26)

The corrections of the form $\bar{B}/\left(k_{B}/r_{B}-\bar{B}\right)$ appear because bindings only happen to unbound receptors, as emphasized in Ref. [5]. However, these nonlinear relations are still hard to implement with simple biochemical components. We solve this by further assuming $\epsilon =\bar{B}/\left(k_{B}/r_{B}\right)\ll 1$, which is true if the receptor is mostly unbound, which happens at low concentrations. This gives

$$c_{c}^{\text{KPR}}\approx \frac{\bar{A}e^{r_{c}T^{c}}r_{c}r_{A}}{k_{c}k_{A}},$$ (27)

$$c_{\text{nc}}^{\text{KPR}}\approx \frac{r_{\text{nc}}}{k_{\text{nc}}}\left(\frac{r_{B}\bar{B}}{k_{B}}-\frac{\bar{A}e^{r_{c}T^{c}}r_{A}}{k_{A}}\right).$$ (28)

These equations are analogous to Eqs (13) and (14). They are easy to realize biochemically (cf. Fig 4(a)): *c*_c_ is related to the concentration of the proofread species A by a rescaling, and *c*_nc_ comes from subtracting rescaled versions of B and A from each other. The subtraction can be done by the third species C, activated by B and suppressed by A. Since *ϵ* ≪ 1, then $\bar{A}$ and $\bar{B}$ are small, and many such activation-suppression schemes are linearized as the subtraction [8]. Note that such incoherent feedforward loops (the receptor activates A and B, which then affect C incoherently by suppressing and activating it, respectively) are ubiquitous in cellular networks downstream of receptors [9].

The bias of $c_{c}^{a}$ and $c_{\text{nc}}^{a}$ due to long, but noncognate binding events, Eq (15), carries over to $c_{c}^{\text{KPR}}$ and $c_{\text{nc}}^{\text{KPR}}$. However, there is an additional contribution since the time to traverse the intermediate states in KPR schemes with multiple intermediate steps is random. Thus *T*^c^ has some variance $\sigma_{T^{c}}^{2}$ [19, 20]. This variability changes the rate of occurrence of long biding events, but they are still rare, nearly independent, and Poisson-distributed. Denoting by 〈⋅〉 the averaging at a fixed *T*^c^, and by $\bar{\cdot }$ the averaging over *T*^c^, we get

$$\bar{\langle n_{l}\rangle }=n\;\bar{P(\tau^{b}>T^{c})}=\frac{n}{\left(k_{c}c_{c}+k_{\text{nc}}c_{\text{nc}}\right)}\bar{\left(k_{c}c_{c}e^{-r_{c}T^{c}}+k_{\text{nc}}c_{\text{nc}}e^{-r_{\text{nc}}T^{c}}\right)}\approx \frac{n\;k_{c}c_{c}\;e^{-r_{c}{\bar{T}}^{c}+\frac{1}{2}r_{c}^{2}\sigma_{T^{c}}^{2}}}{\left(k_{c}c_{c}+k_{\text{nc}}c_{\text{nc}}\right)},$$ (29)

where we have used the approximation ${\bar{T}}^{c}\gg 1/r_{\text{nc}}$ in the last step.

Thus $\sigma_{T^{c}}^{2}$ effectively renormalizes the cutoff to ${\bar{T}}^{c}-\frac{1}{2}r_{c}\sigma_{T^{c}}^{2}$. Replacing *T*^c^ in Eqs (27) and (28) by its renormalized value, which is an easy change in the scaling factors, removes this additional bias due to the random *T*^c^ in the KPR scheme.

Since long bindings are rare, the variance of the KPR estimator is dominated again generally by $\bar{A}$, but not $\bar{B}$. The intrinsic stochasticity in the production of molecules of A contributes to the variance. However, this contribution can be made arbitrarily small by increasing *k*_A_, and we neglect it here. A larger contribution comes from the random number of long bound intervals and a random duration of each of them. To calculate this, in the limit of rare long binding events, we use well-known results in the theory of noise propagation in chemical networks [21]

$$\frac{\sigma_{A}^{2}}{{\bar{A}}^{2}}\approx \frac{\left(1+k_{c}c_{c}/r_{c}+k_{\text{nc}}c_{\text{nc}}/r_{\text{nc}}\right)e^{r_{c}T^{c}-\frac{1}{2}r_{c}^{2}\sigma_{T^{c}}^{2}}}{k_{c}c_{c}\left(1/r_{c}+1/r_{A}\right)}=\frac{e^{r_{c}T^{c}-\frac{1}{2}r_{c}^{2}\sigma_{T^{c}}^{2}}}{k_{c}c_{c}\left(1/r_{c}+1/r_{A}\right)}+O\left(\epsilon \right).$$ (30)

This is a direct analog of Eq (16).

In principle, one can measure more than two concentrations similarly, as long as all species have distinct off-rates. For example, to estimate three concentrations, one needs an additional branch downstream of the receptor that proofreads for an intermediate time. Then the branches with the strongest, intermediate, and no proofreading would measure approximately the highest affinity ligand, a combination of the two higher affinity ligands, and all three ligands, respectively. Appropriate activation and inhibition of downstream targets will then allow identifying individual concentrations from these combined readouts. The error (the variance of the ML estimator, and both the bias and the variance for the approximate and the KPR estimators) would grow with an increasing number of ligand species, largely because a larger range of off-rates would be required to disambiguate more ligands. However, this would still represent a dramatic increase in the information gained by a receptor that tracks its precise temporal dynamics, rather than just the average binding state.

### Using precise timing to disambiguate two similar ligands

Here we depart slightly from our scenario and show how a KPR-based scheme relying on the entire temporal sequence of activation / deactivation events can estimate the concentration of a *single* cognate ligand even if the two ligands have very similar off-rates *r*_c_ ≲ *r*_nc_, a situation common in immunology. In such a situation, the KPR branch gets activated not just by the cognate ligand, but also by the non-cognate ligand (though at a smaller rate). When the goal is the accurate estimation of the cognate ligand only, then the contribution to the KPR branch by the non-cognate ligand needs to be removed. To construct a signal transduction network able to do this, we abstract from the existing detailed model of Fc*ϵ*RI immunological receptor [9], a well studied eukaryotic signal transduction system mediating many allergic reactions [22]. Here the main signaling branch gets activated through the Lyn-Syk kinase pathway following kinetic proofreading after a ligand binds to the receptor [9]. However, receptor binding excites an additional branch early on, after only one step in kinetic proofreading (a single phosphorylation on the *β* chain of the receptor). This branch activates Inpp5d (SHIP) phosphotase, which later dephosphorylates Phosphatidylinositol 3-phosphate (PIP3), a key downstream output of the main signaling branch, and sequesters the dephosphorylated product PtdIns(3, 4)P_2_ [9]. The part of this signaling motif relevant for our analysis is summarized in a deliberately simplified signaling diagram in Fig 4(b), where A stands for PtdIns(3, 4, 5)P_3_ (PIP3), I stands for PtdIns(3, 4)P_2_, and I is produced by SHIP. Further, R is the Fc*ϵ*RI receptor bound to an antibody, and cognate and noncognate molecules are the antigens specific/nonspecific to the bound antibody.

In this network, we consider the main activator branch (A), activated after the usual KPR delay, and hence sensitive to long binding events only (which now have contributions both from *k*_c_ and *k*_nc_). The secondary inhibiting branch (I) is activated by many more binding events, though the shortest, nonspecific background binding events may be removed from both branches by additional proofreading steps (an early cross-phosphorylation event in the Fc*ϵ*RI system). The messengers in both branches later form a complex AI, and only A not in the complex activates further downstream signaling. If the production rates of A and I are appropriately matched (which can be done if the off-rates are known *a priori*, which they should be for such a molecular signal detection system), this sequestration of A by I can effectively remove the contribution to the A branch coming from the non-cognate ligand. The kinetic diagram can be described with the following rate equations (where, for simplicity, we neglect the first proofreading common to both branches):

$$\frac{dA}{dt}=\beta_{A}-r_{A}A-r_{\text{AI}}AI,$$ (31)

$$\frac{dI}{dt}=\beta_{I}-r_{I}I-r_{\text{AI}}AI,$$ (32)

where *r*_A/I_ are the degradation rates of the messengers A and I, *r*_AI_ is the sequestration rate, and *β*_A/I_ are the messenger production rates, derived as above:

$$\beta_{A}=\frac{k_{c}c_{c}/r_{c}e^{-r_{c}T^{c}}+k_{\text{nc}}c_{\text{nc}}/r_{\text{nc}}e^{-r_{\text{nc}}T^{c}}}{1+k_{c}c_{c}/r_{c}+k_{\text{nc}}c_{\text{nc}}/r_{\text{nc}}}k_{A},$$ (33)

$$\beta_{I}=\frac{k_{c}c_{c}/r_{c}+k_{\text{nc}}c_{\text{nc}}/r_{\text{nc}}}{1+k_{c}c_{c}/r_{c}+k_{\text{nc}}c_{\text{nc}}/r_{\text{nc}}}k_{I}.$$ (34)

Here *k*_A/I_ are the rates of production of A and I, respectively, when the receptor has been bound for a sufficiently long time to produce either.

We assume for simplicity *r*_A_ = *r*_I_. Further, we choose *r*_A_ = *r*_I_ ≪ *r*_AI_*A* ∼ *r*_AI_*I*, so that sequestration rather than degradation is primarily responsible for the disappearance of the messengers. Then the steady state solution of the rate equations (Eqs 31 and 32) is [23] (see Methods):

$${\bar{A}}_{\text{ss}}=\frac{\left(\beta_{A}-\beta_{I}\right)}{2r_{I}}+\sqrt{{\left(\frac{\beta_{A}-\beta_{I}}{2r_{I}}\right)}^{2}+\frac{\beta_{A}}{r_{\text{AI}}}},$$ (35)

$${\bar{I}}_{\text{ss}}=\frac{\left(\beta_{I}-\beta_{A}\right)}{2r_{I}}+\sqrt{{\left(\frac{\beta_{I}-\beta_{A}}{2r_{I}}\right)}^{2}+\frac{\beta_{I}}{r_{\text{AI}}}}.$$ (36)

The numerators of both *β*_A_ and *β*_I_ are linear combinations of *c*_c_ and *c*_nc_. If the parameters of the biochemical networks are such that the production rate of the proofread branch is $k_{A}=k_{I}e^{r_{\text{nc}}T^{c}}$, then $\left(\beta_{\text{A}}-\beta_{\text{I}}\right)=\frac{k_{\text{c}}c_{\text{c}}/r_{\text{c}}\left(e^{\left(r_{\text{nc}}-r_{\text{c}}\right)T^{\text{c}}}-1\right)}{\left(1+k_{\text{c}}c_{\text{c}}/r_{\text{c}}+k_{\text{nc}}c_{\text{nc}}/r_{\text{nc}}\right)}k_{\text{I}}>0$, which has a *c*_nc_-independent numerator. Thus the contribution of non-cognate ligand to the activator branch is largely sequestered. Moreover, for large *r*_AI_, we have ${\bar{A}}_{\text{ss}}\propto {\left(1+k_{c}c_{c}/r_{c}+k_{\text{nc}}c_{\text{nc}}/r_{\text{nc}}\right)}^{-1}$, so that the activation of the A branch *decreases* as *c*_nc_ increases. In contrast, if *c*_c_ = 0 (no cognate ligands present), then ${\bar{A}}_{\text{ss}}={\bar{I}}_{\text{ss}}=\sqrt{\frac{k_{I}}{r_{\text{AI}}}\frac{k_{\text{nc}}c_{\text{nc}}}{k_{\text{nc}}c_{\text{nc}}+r_{\text{nc}}}}$, which grows with *c*_nc_. This behavior is reminiscent of the agonist-antagonist picture in Fc*ϵ*RI receptor activation [24]: a weak ligand by itself can activate the cellular response, but it inhibits (antagonizes) activation of the response by a stronger agonist if both are present.

## Discussion

The realization of Refs. [5, 13, 12] and others that the detailed temporal sequence of binding and unbinding events carries more information about the ligand concentration than the mean receptor occupancy is a conceptual breakthrough. It parallels the realization in the computational neuroscience community that precise timing of spikes carries more information about the stimulus than the mean neural firing rate [25–30], and it has a potential to be equally impactful. This extra information when measuring one ligand concentration with one receptor [5, 12] amounted to increasing the sensing accuracy by a constant prefactor, or, equivalently, getting only a finite number of additional bits from even a very long measurement [31]. In contrast, here we show that two concentrations can be measured with one receptor with the variance that decreases inversely proportionally to the number of observations, *n*, Eq (16), or to the integration time, 1/*r*_B_, Eq (30), so that the accuracy is only an (often small) prefactor lower than would be possible with one receptor per ligand species. Asymptotically, this doubles the information obtained by the receptor [31].

Crucially, such improvement would not be possible without the cross-talk, or binding among noncognate ligands and receptors. Normally, the cross-talk is considered a nuisance that must be suppressed [32, 33]. Instead, we argue that cross-talk can be beneficial by recruiting more receptor types to measure the concentration of the same ligand. In particular, this allows having fewer receptor than ligand species, potentially illuminating how cells function reliably in chemically complex environments with few receptor types. Further, the cross-talk can increase the dynamic range of the entire system: a ligand may saturate its cognate receptor, preventing accurate measurement of its (high) concentration, but it may be in the sensitive range of non-cognate receptors at the same time. Finally, the increased bandwidth may lead to improvements in sensing a time-dependent ligand concentration [11, 13]. In forthcoming publications, we plan to explore such many-to-many sensory schemes, extending ideas of Ref. [34] to tracking temporal sequences of activation of the receptor and to temporally varying environments.

While the exact maximum likelihood inference of multiple concentrations from a temporal binding-unbinding sequence is rather complex, we showed that when the cognate and the non-cognate off-rates are substantially different, there is a simpler, approximate, but accurate inference procedure for joint measurements of cognate and noncognate ligands. And even if the off-rates are close, one can still measure the cognate ligand concentration reliably. Crucially, this inference can be performed by biochemical motifs readily available to the cell. Namely, one needs two branches of activation downstream of the receptor, with at least one of them having a kinetic proofreading (KPR) time delay. Then the individual ligand concentrations can be obtained by mutual inhibition between the two branches, or by incoherent feedforward loops. We emphasize again that this allows estimation of *multiple* concentrations from activity of a single receptor, in contrast to a better estimation of just one concentration [12].

Our simple models only illustrate a wide class of models that can use the temporal structure of the receptor binding sequence to measure more that one ligand concentrations for various ligand combinations, including similar and dissimilar ligands. Additional branches from different points in the proofreading cascade provide additional information about the binding affinities of the mixture of ligands present in the environment, and then algebraic operations on these readouts can be performed by a large diversity of feedforward and feedback loops, competitions for the substrate and the enzyme, and so on. For example, in our simple model, the action of the antagonist is due to the competition for available receptors, while experiments suggest competition for a critical initiating kinase [24], which would require a straightforward modification of the model. Similarly, antagonists are usually “medium” affinity ligands, while very weak ligands do not antagonize receptors. As illustrated in Fig 4(b), this can be achieved by having an additional KPR time delay common to both A and I branches, which occurs in practice [9].

The kinetic diagram for the Fc*ϵ*RI receptor is not unique, and similar (though not equivalent) structures exist for other immune cells and receptors as well [9]. Such common structural features result in a similar phenomenology of activation profiles, which are different for pure ligands and ligand mixtures, and depend nontrivially on the details of the binding affinities and concentrations of the ligands in the mixture [10, 16, 35–38]. Interestingly, on longer time scales, a potentially related phenomenon in innate immune response is that of endotoxin tolerance (desensitization to commonly present ligands) [39], which also affects ligands of different affinity differently, and in this case also depends on the history of exposure to other ligands [40]. It is mediated by SHIP, a crucial player in our analysis of Fc*ϵ*RI signaling [41], whose activity may be interpreted as setting the relative gain on the A and I branches of Fig 4(b), thus resulting in a more accurate signal estimation. In other words, one interpretation of the known results is that, as various feedback loops increase the activity of SHIP in response to frequent activation of signaling downstream of the receptor, the amount of I increases, thus sequestering more A, lowering its steady-state activity, and inducing tolerance. An important contribution of the understanding developed here is that one can try to interpret these various kinetic diagrams and their phenomenological consequences as implementing estimation of concentrations of potentially many ligands (rather detection of a single one [11, 13, 16]), and maybe even doing it in a (nearly) Maximum Likelihood optimal fashion, under various assumptions about the number of distinct ligands, their relative abundance, and the (dis)similarity of the off-rates. Exploring feasibility of such an interpretation is an additional interesting venue for future research.

In summary, monitoring precise temporal sequences of receptor activation/deactivation opens up new and exciting possibilities for environment sensing by cells.

## Methods

Here we provide mathematical derivations of some of the steps ommitted in the *Results*.

### Derivation of maximum likelihood equations

We start with:

$$P\equiv P\left(\left\{\tau_{i}^{b},\;\tau_{i}^{u}\right\}|c_{c},c_{\text{nc}}\right)=\frac{1}{Z}\prod_{i=1}^{n}\left[e^{-\tau_{i}^{u}\left(k_{c}c_{c}+k_{\text{nc}}c_{\text{nc}}\right)}\left(k_{c}c_{c}\;r_{c}\;e^{-\tau_{i}^{b}r_{c}}+k_{\text{nc}}c_{\text{nc}}\;r_{\text{nc}}\;e^{-\tau_{i}^{b}r_{\text{nc}}}\right)\right].$$ (37)

The log-likelihood of *k*_c,nc_ is the logarithm of *P*:

$$\log\left(P\right)=-\log Z-\sum_{i=1}^{n}\left[\tau_{i}^{u}\left(k_{c}c_{c}+k_{\text{nc}}c_{\text{nc}}\right)+\log\left(k_{c}c_{c}\;r_{c}\;e^{-\tau_{i}^{b}r_{c}}+k_{\text{nc}}c_{\text{nc}}\;r_{\text{nc}}\;e^{-\tau_{i}^{b}r_{\text{nc}}}\right)\right].$$ (38)

Taking the derivatives of the log-likelihood w. r. t. *c*_c_ and *c*_nc_ and setting them to zero gives the Maximum Likelihood (ML) equations for the concentrations. These are:

$$\frac{\partial \log(P)}{\partial c_{c}}=-\sum_{i=1}^{n}\tau_{i}^{u}k_{c}+\sum_{i=1}^{n}\frac{k_{c}r_{c}e^{-\tau_{i}^{b}r_{c}}}{D(c_{c}^{*},c_{\text{nc}}^{*},\tau_{i}^{b})}=0,$$ (39)

$$\frac{\partial \log(P)}{\partial c_{\text{nc}}}=-\sum_{i=1}^{n}\tau_{i}^{u}k_{\text{nc}}+\sum_{i=1}^{n}\frac{k_{\text{nc}}r_{\text{nc}}e^{-\tau_{i}^{b}r_{\text{nc}}}}{D(c_{c}^{*},c_{\text{nc}}^{*},\tau_{i}^{b})}=0.$$ (40)

Here, $D\left(c_{c}^{*},c_{\text{nc}}^{*},\tau_{i}^{b}\right)=(k_{c}c_{c}^{*}\;r_{c}\;e^{-\tau_{i}^{b}r_{c}}+k_{\text{nc}}c_{\text{nc}}^{*}\;r_{\text{nc}}\;e^{-\tau_{i}^{b}r_{\text{nc}}})$, with * denoting the ML solution.

Denoting by $T^{u}=\sum_{i=1}^{n}\tau_{i}^{u}$, the total time for which the receptor is unbound, these equations can be rewritten as

$$-k_{c}T^{u}+\sum_{i=1}^{n}\frac{k_{c}r_{c}e^{-\tau_{i}^{b}r_{c}}}{D(k_{c}^{*},k_{\text{nc}}^{*},\tau_{i}^{b})}=0,$$ (41)

$$-k_{\text{nc}}T^{u}+\sum_{i=1}^{n}\frac{k_{\text{nc}}r_{\text{nc}}e^{-\tau_{i}^{b}r_{\text{nc}}}}{D(k_{c}^{*},k_{\text{nc}}^{*},\tau_{i}^{b})}=0.$$ (42)

Multiplying Eqs (41) and (42) by $c_{c}^{*}$ and $c_{\text{nc}}^{*}$, respectively, and adding them gives

$$k_{c}c_{c}^{*}+k_{\text{nc}}c_{\text{nc}}^{*}=\frac{n}{T^{u}}.$$ (43)

### Comparison of simulations with analytical results for single concentration estimation

**Fig 5:**
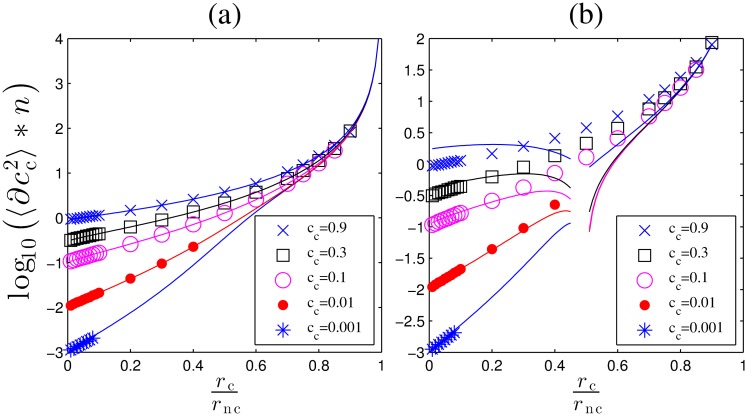
Comparison of simulations with analytical results for single concentration estimation. (a) The variance of the estimated concentration in simulations (markers) matches quite well with the integral expression, Eq. (7) in Ref. [12]. (b) Comparison of simulations to analytical expression derived for low concentrations in Eq. (9) of Ref. [12].

Here we compare the results obtained from the numerical simulations to the analytical expressions derived in Ref. [12] for detection of the concentration of the cognate ligand in a background of spurious ligands. The variance of the concentration estimation obtained from the simulations matches quite well with integral expression, Eq. (7) in Ref. [12], Fig 5a. Note that this expression is inverse of the (1, 1) term of the Hessian matrix, Eq 7. The analytical results obtained for the low concentration of the cognate ligand compared to a non cognate ligand (*c*_c_ ≪ *c*_nc_) also match the simulations, Fig 5b.

### Approximate solution

#### Derivation of Eqs 13 and 14

Defining long events as $\tau_{i}^{b}\geq T^{c}$ and using Eq (43), we rewrite Eq (41) as

$$\frac{k_{c}n}{k_{c}c_{c}^{*}+k_{\text{nc}}c_{\text{nc}}^{*}}=\left(\sum_{\tau_{i}^{b}\geq T^{c}}+\sum_{\tau_{i}^{b}<T^{c}}\right)\frac{k_{c}r_{c}e^{-\tau_{i}^{b}r_{c}}}{\left(k_{c}c_{c}^{*}\;r_{c}\;e^{-\tau_{i}^{b}r_{c}}+k_{\text{nc}}c_{\text{nc}}^{*}\;r_{\text{nc}}\;e^{-\tau_{i}^{b}r_{\text{nc}}}\right)}.$$ (44)

Assuming that all long events are cognate, *T*^c^ ≫ 1/*r*_nc_, we can ignore the $k_{\text{nc}}c_{\text{nc}}^{*}\;r_{\text{nc}}\;e^{-\tau_{i}^{b}r_{\text{nc}}}$ in the denominator in the first sum. This gives

$$\frac{k_{\text{c}}n}{\left(k_{\text{c}}c_{\text{c}}^{a}+k_{\text{nc}}c_{\text{nc}}^{a}\right)}=\frac{n_{\text{l}}}{c_{\text{c}}^{\text{a}}}+\underset{\tau_{i}^{\text{b}}<T^{\text{c}}}{\sum }\frac{k_{\text{c}}r_{\text{c}}e^{-\tau_{i}^{\text{b}}r_{\text{c}}}}{D\left(c_{\text{c}}^{\text{a}},c_{\text{nc}}^{\text{a}},\tau_{i}^{\text{b}}\right)},$$

where *n*_l_ is the number of long events, and the superscript “a” stands for the *a*pproximate solution. If further *T* is long enough so that there are many short events, and a single binding duration hardly affects $k_{c}^{*}$, then the sum in Eq (9) can be approximated by the expectation value:

$$\frac{n}{\left(k_{c}c_{c}^{a}+k_{\text{nc}}c_{\text{nc}}^{a}\right)}=\frac{n_{l}}{k_{c}c_{c}^{a}}+\left(n-n_{l}\right)\int_{0}^{T_{c}}\frac{r_{c}e^{-\tau^{b}r_{c}}P\left(\tau^{b}|c_{c}^{a},c_{\text{nc}}^{a}\right)d\tau^{b}}{D(c_{c}^{a},c_{\text{nc}}^{a},\tau^{b})},$$ (45)

where $P(\tau^{b}|c_{c}^{a},c_{\text{nc}}^{a})$ is the probability of observing a binding event of duration *τ*^b^ for the given binding rates,

$$P\left(\tau^{b}|c_{c}^{a},c_{\text{nc}}^{a}\right)=\frac{D(c_{c}^{a},c_{\text{nc}}^{a},\tau^{b})}{\left(k_{c}c_{c}^{a}+k_{\text{nc}}c_{\text{nc}}^{a}\right)}.$$ (46)

Plugging Eq (11) into Eq (10), we obtain

$$\frac{n}{\left(k_{c}c_{c}^{a}+k_{\text{nc}}c_{\text{nc}}^{a}\right)}=\frac{n_{l}}{k_{c}c_{c}^{a}}+\left(n-n_{l}\right)\int_{0}^{T_{c}}\frac{r_{c}e^{-\tau^{b}r_{c}}d\tau^{b}}{\left(k_{c}c_{c}^{a}+k_{\text{nc}}c_{\text{nc}}^{a}\right)},$$ (47)

which gives:

$$\frac{1}{\left(k_{c}c_{c}^{a}+k_{\text{nc}}c_{\text{nc}}^{a}\right)}=\frac{n_{l}}{nk_{c}c_{c}^{a}}+\left(1-\frac{n_{l}}{n}\right)\frac{1-e^{-r_{c}T^{c}}}{\left(k_{c}c_{c}^{a}+k_{\text{nc}}c_{\text{nc}}^{a}\right)}.$$ (48)

Assuming *n*_l_ ≪ *n*, we get:

$$\frac{1}{\left(k_{c}c_{c}^{a}+k_{\text{nc}}c_{\text{nc}}^{a}\right)}=\frac{n_{l}}{nk_{c}c_{c}^{a}}+\frac{1}{\left(k_{c}c_{c}^{a}+k_{\text{nc}}c_{\text{nc}}^{a}\right)}-\frac{e^{-r_{c}T^{c}}}{\left(k_{c}c_{c}^{a}+k_{\text{nc}}c_{\text{nc}}^{a}\right)}.$$ (49)

This gives,

$$c_{c}^{a}=\frac{1}{k_{c}}\frac{n_{l}}{T^{u}}e^{r_{c}\;T^{c}}.$$ (50)

Finally, using Eq 43 we get

$$c_{\text{nc}}^{a}=\frac{1}{k_{\text{nc}}}\left(\frac{n}{T^{u}}-\frac{n_{l}}{T^{u}}e^{r_{c}\;T^{c}}\right).$$ (51)

#### Error in approximate solution at $T_{*}^{c}$

Plugging Eq 20 in Eq 15 we get,

$$\begin{array}{cc} \langle c_{c}^{a}\rangle & \approx c_{c}+\frac{k_{\text{nc}}c_{\text{nc}}}{k_{c}}e^{-\frac{\left(r_{\text{nc}}-r_{c}\right)}{\left(2r_{\text{nc}}-r_{c}\right)}\log\left[2T^{u}\left(\frac{r_{\text{nc}}}{r_{c}}-1\right)\frac{k_{\text{nc}}^{2}c_{\text{nc}}^{2}}{k_{c}c_{c}}\right]}\\ & =c_{c}+\frac{k_{\text{nc}}c_{\text{nc}}}{k_{c}}{\left[2T^{u}\left(\frac{r_{\text{nc}}}{r_{c}}-1\right)\frac{k_{\text{nc}}^{2}c_{\text{nc}}^{2}}{k_{c}c_{c}}\right]}^{-\frac{\left(r_{\text{nc}}-r_{c}\right)}{\left(2r_{\text{nc}}-r_{c}\right)}} \end{array}$$ (52)

In the limit *r*_nc_ >> *r*_c_, the second term goes as $\sqrt{c_{c}}$.

Now using this in Eq 16, we get:

$$\begin{array}{c} \sigma^{2}\left(c_{c}^{a}\right)\approx \langle c_{c}^{a}\rangle \;\frac{\left(c_{c}+k_{\text{nc}}c_{\text{nc}}/k_{c}\right)}{n}e^{\frac{r_{c}}{\left(2r_{\text{nc}}-r_{c}\right)}\log\left[2T^{u}\left(\frac{r_{\text{nc}}}{r_{c}}-1\right)\frac{k_{\text{nc}}^{2}c_{\text{nc}}^{2}}{k_{c}c_{c}}\right]}\\ =\left(c_{c}+\frac{k_{\text{nc}}c_{\text{nc}}}{k_{c}}{\left[2T^{u}\left(\frac{r_{\text{nc}}}{r_{c}}-1\right)\frac{k_{\text{nc}}^{2}c_{\text{nc}}^{2}}{k_{c}c_{c}}\right]}^{-\frac{\left(r_{\text{nc}}-r_{c}\right)}{\left(2r_{\text{nc}}-r_{c}\right)}}\right)\frac{\left(c_{c}+k_{\text{nc}}c_{\text{nc}}/k_{c}\right)}{n}{\left[2T^{u}\left(\frac{r_{\text{nc}}}{r_{c}}-1\right)\frac{k_{\text{nc}}^{2}c_{\text{nc}}^{2}}{k_{c}c_{c}}\right]}^{\frac{r_{c}}{\left(2r_{\text{nc}}-r_{c}\right)}}. \end{array}$$ (53)

### Kinetic proofreading for approximate estimation: Derivation of Eqs (21) and (22)

In the biochemical network in Fig 4(a) of the main text, the receptor R activates two messenger molecules, A and B. The former is activated with the rate *k*_A_ only if the receptor stays bound for longer than a certain *T*^c^ (with the delay achieved using the KPR intermediate states). The latter is activated with the rate *k*_B_ whenever the receptor is bound. The molecules deactivate with the rates *r*_A_ and *r*_B_, respectively, and all activations/deactivations are first-order reactions. The rate equation for the two molecules can be written as:

$$\frac{dA}{dt}=k_{A}\Theta \left(\tau_{b}>T^{c}\right)-r_{A}A,$$ (54)

$$\frac{dB}{dt}=k_{B}\Theta \left(\tau_{b}>0\right)-r_{B}B.$$ (55)

The Θ functions represent the fact that *A* is produced only when the receptor has been bound for longer than the cutoff time *T*^*c*^, and *B* is produced only when the receptor is bound.

The steady state value of $\bar{A}$ can be obtained by equating the average deactivation rate $r_{A}\bar{A}$ to *k*_A_ times the fraction of time the receptor occupancy was larger than the cutoff, *T*^*c*^, i.e.,

$$r_{A}\bar{A}=k_{A}\frac{\langle \tau_{b}>T^{c}\rangle }{\langle \tau_{u}\rangle +\langle \tau_{b}\rangle }.$$ (56)

Similarly, $\bar{B}$ can be obtained as:

$$r_{B}\bar{B}=k_{B}\frac{\langle \tau_{b}\rangle }{\langle \tau_{u}\rangle +\langle \tau_{b}\rangle }.$$ (57)

Therefore, the mean concentrations of the messenger molecules are:

$$\bar{A}=\frac{k_{c}c_{c}/r_{c}e^{-r_{c}T^{c}}+k_{\text{nc}}c_{\text{nc}}/r_{\text{nc}}e^{-r_{\text{nc}}T^{c}}}{1+k_{c}c_{c}/r_{c}+k_{\text{nc}}c_{\text{nc}}/r_{\text{nc}}}\frac{k_{A}}{r_{A}},$$ (58)

$$\bar{B}=\frac{k_{c}c_{c}/r_{c}+k_{\text{nc}}c_{\text{nc}}/r_{\text{nc}}}{1+k_{c}c_{c}/r_{c}+k_{\text{nc}}c_{\text{nc}}/r_{\text{nc}}}\frac{k_{B}}{r_{B}}.$$ (59)

### Using precise timing to disambiguate two close ligands: Derivation of Eqs (35) and (36)

The rate equations are:

$$\frac{dA}{dt}=\beta_{A}-r_{A}A-r_{\text{AI}}AI,$$ (60)

$$\frac{dI}{dt}=\beta_{I}-r_{I}I-r_{\text{AI}}AI.$$ (61)

Equating the r. h. s. to zero gives the steady state conditions:

$$\beta_{A}-r_{A}{\bar{A}}_{\text{ss}}-r_{\text{AI}}{\bar{A}}_{\text{ss}}{\bar{I}}_{\text{ss}}=0,$$ (62)

$$\beta_{I}-r_{I}{\bar{I}}_{\text{ss}}-r_{\text{AI}}{\bar{A}}_{\text{ss}}{\bar{I}}_{\text{ss}}=0.$$ (63)

The latter of these can be rewritten as:

$${\bar{I}}_{\text{ss}}=\frac{\beta_{I}}{r_{I}+r_{\text{AI}}{\bar{A}}_{\text{ss}}}.$$ (64)

Plugging this in Eq (62), we get

$$\beta_{A}-r_{A}{\bar{A}}_{\text{ss}}-\frac{r_{\text{AI}}{\bar{A}}_{\text{ss}}\beta_{I}}{r_{I}+r_{\text{AI}}{\bar{A}}_{\text{ss}}}=0,$$ (65)

which can be simplified to:

$${\bar{A}}_{\text{ss}}^{2}+\left(\frac{r_{I}}{r_{\text{AI}}}+\frac{\left(\beta_{I}-\beta_{A}\right)}{r_{A}}\right){\bar{A}}_{\text{ss}}-\frac{\beta_{A}r_{I}}{r_{\text{AI}}r_{A}}=0.$$ (66)

This quadratic equation has the solution:

$${\bar{A}}_{\text{ss}}=-\left(\frac{r_{\text{I}}}{2r_{\text{AI}}}+\frac{\left(\beta_{\text{I}}-\beta_{\text{A}}\right)}{2r_{\text{A}}}\right)+\sqrt{{\left(\frac{r_{\text{I}}}{2r_{\text{AI}}}+\frac{\left(\beta_{\text{I}}-\beta_{\text{A}}\right)}{2r_{\text{A}}}\right)}^{2}+\frac{\beta_{\text{A}}r_{\text{I}}}{r_{\text{AI}}r_{\text{A}}}.}$$ (67)

Now sssuming *r*_A_ = *r*_I_ and *r*_A_ = *r*_I_ ≪ *r*_AI_*A* ∼ *r*_AI_*I*, we get:

$${\bar{A}}_{\text{ss}}=\frac{\left(\beta_{\text{A}}-\beta_{\text{I}}\right)}{2r_{\text{I}}}+\sqrt{{\left(\frac{\beta_{\text{I}}-\beta_{\text{A}}}{2r_{\text{A}}}\right)}^{2}+\frac{\beta_{\text{A}}}{r_{\text{AI}}}}$$ (68)

One can similarly can get the equation for ${\bar{I}}_{\text{ss}}$ as well.
